# Structural and Electrical Remodeling of the Sinoatrial Node in Diabetes: New Dimensions and Perspectives

**DOI:** 10.3389/fendo.2022.946313

**Published:** 2022-07-07

**Authors:** Lina T. Al Kury, Stephanie Chacar, Eman Alefishat, Ali A. Khraibi, Moni Nader

**Affiliations:** ^1^ Department of Health Sciences, College of Natural and Health Sciences, Zayed University, Abu Dhabi, United Arab Emirates; ^2^ Department of Physiology and Immunology, College of Medicine and Health Sciences, Khalifa University of Science and Technology, Abu Dhabi, United Arab Emirates; ^3^ Department of Pharmacology, College of Medicine and Health Sciences, Khalifa University of Science and Technology, Abu Dhabi, United Arab Emirates; ^4^ Department of Biopharmaceutics and Clinical Pharmacy, School of Pharmacy, The University of Jordan, Amman, Jordan; ^5^ Center for Biotechnology, Khalifa University of Science and Technology, Abu Dhabi, United Arab Emirates

**Keywords:** sinoatrial node, diabetes, action potential, ion channels, gap junctions, structural remodeling, metabolic changes

## Abstract

The sinoatrial node (SAN) is composed of highly specialized cells that mandate the spontaneous beating of the heart through self-generation of an action potential (AP). Despite this automaticity, the SAN is under the modulation of the autonomic nervous system (ANS). In diabetes mellitus (DM), heart rate variability (HRV) manifests as a hallmark of diabetic cardiomyopathy. This is paralleled by an impaired regulation of the ANS, and by a pathological remodeling of the pacemaker structure and function. The direct effect of diabetes on the molecular signatures underscoring this pathology remains ill-defined. The recent focus on the electrical currents of the SAN in diabetes revealed a repressed firing rate of the AP and an elongation of its tracing, along with conduction abnormalities and contractile failure. These changes are blamed on the decreased expression of ion transporters and cell-cell communication ports at the SAN (i.e., HCN4, calcium and potassium channels, connexins 40, 45, and 46) which further promotes arrhythmias. Molecular analysis crystallized the RGS4 (regulator of potassium currents), mitochondrial thioredoxin-2 (reactive oxygen species; ROS scavenger), and the calcium-dependent calmodulin kinase II (CaMKII) as metabolic culprits of relaying the pathological remodeling of the SAN cells (SANCs) structure and function. A special attention is given to the oxidation of CaMKII and the generation of ROS that induce cell damage and apoptosis of diabetic SANCs. Consequently, the diabetic SAN contains a reduced number of cells with significant infiltration of fibrotic tissues that further delay the conduction of the AP between the SANCs. Failure of a genuine generation of AP and conduction of their derivative waves to the neighboring atrial myocardium may also occur as a result of the anti-diabetic regiment (both acute and/or chronic treatments). All together, these changes pose a challenge in the field of cardiology and call for further investigations to understand the etiology of the structural/functional remodeling of the SANCs in diabetes. Such an understanding may lead to more adequate therapies that can optimize glycemic control and improve health-related outcomes in patients with diabetes.

## 1 The Sinoatrial Node as a Pacemaker of the Heart

Cardiac rhythm is controlled by the activity of a heterogeneous collection of highly specialized cells forming the SAN, located at the wall (epicardium) of the right atrium, laterally to the entrance of the superior vena cava, and in the vicinity to the cristae terminalis (1, 2). Since its discovery about a century ago (1907) by Arthur Keith and Martin Flack, the SAN has attracted the attention of scientists to decipher its characteristics (3).

The length of the SAN ranges from 8-21.5 mm, comprising the head, the central area and the tail (4, 5). Optical mapping techniques identified several spots, within the SAN, that discharge action potentials (APs), ultimately leading to the depolarization of the atrial muscle (6). The unique electrophysiological properties of the SAN cells (SANCs) are conducive to self-excitation yielding spontaneous depolarization, thus pacemaker activity, in the absence of external stimuli (7). This is mandated by the ingress of sodium (funny current) and calcium ions through the sarcolemma (8) which constantly lifts up the unstable resting membrane potential (~ 60 mV) of SANCs towards the threshold (~ -40 mV), before the upstroke of the AP arises (9, 10). Despite this, the self-excitation process *per se* is insufficient; it still requires a special cellular organization/structure that promotes the exit of the AP throughout well-defined pathways to spread through, and efficiently depolarize the adjacent myocardium (left atrium) (11). These include a remarkable cell-cell communication profile implicating low conductance gap junctions with a particular transmission pattern of the electrical signals, along with an electrical insulator (connective tissue) surrounding the grouped SANCs (12). The connective tissue is needed to avoid the random dissipation of the depolarization wave and to focus its trajectory through specific exit pathways for an effective propagation of the AP through the atrial myocardium (13). The blood supply to the SAN is mainly through the right coronary artery in most cases, to a lesser extent by the circumflex of the left coronary artery, and by both coronary arteries in a very minor portion of the population (14–16). Therefore, the safety of cardiac rhythm highly hinges on a properly functioning SAN that must be constantly supplied by the appropriate amount of oxygen/nutrients.

## 2 SAN Cells Lineage and Developmental Changes

SANCs are structurally and functionally different from the surrounding cardiomyocytes (17, 18). The SAN comprises a collection of weakly connected cells (pacemaker cells, adipocytes, myocytes, and fibroblasts) out of which those defining the pacemaker activity of the node are divided into three main types of cells; the elongated spindle shaped cells (~80 µm in length), the spindle cells (~ 40µm), and the spider-shaped cells [reviewed in (19)]. A careful inspection of human SAN revealed the presence of mainly three different types of cells: the pale (P) cells organized in clusters with elongated cytoplasmic extensions, the transitional (T) cells that resemble cardiomyocytes but with fewer sarcomeres, and the fibroblast-like cells with long bi-tripolar contacting cells (20). All together, these cells are insulated by fibrous tissues from the rest of the atrial myocardium. This insulation shields the SANCs from atrial hyperpolarization and provides a unidirectional route for the depolarization wave initiated at the SAN center to spread in mainly three directions (outside the superior vena cava, outside the inferior vena cava, and between both venae cava) and therefore to invade the atrial muscle (21). Despite the heterogeneity of these cells, along with the difference in their distribution across the SAN (cristae terminals vs atrial septum), there are unifying active electrical properties at the plasma membrane that support the pacing activity under normal circumstances. At the initial stages of cardiac development, all cardiac cells possess pacemaker activity, however, the majority develop into working myocardium and few cells form the conduction system of the heart (SAN, Atrioventricular node, and His-Purkinje fibers). This is achieved by localized and targeted repression of differentiation of specific genes that drive these cells into cardiac muscle *via* an interplay between different modulators of the transcription (example: Tbx5, Nkx-5, Tbx-2, Tb-3, and Id2) (22). A primordium SAN is shown as early as Embryonic (E) day 10.5 in mouse hearts (23). At this stage, SANCs are relatively poor in organelles and myofibrils compared to the other cardiomyocytes at E16 and E18 in mice, and they are characterized by a strikingly poor proliferative capacity at embryonic levels (compared with cardiomyocytes) (24). At birth, the pacing of the SAN overrides all other parts of the conduction system (Atrioventricular node and His-Purkinje fibers) and guarantees a heart rate of about 70-90 beats per min in healthy subjects. Heart rate variability (HRV) is tightly linked to changes in the activity of the SAN (intrinsic or extrinsic factors). With age, the decrease in the volume of nodal cells and SAN tissues, along with the development of fibrosis can result in dysfunction of the SAN (25, 26).

## 3 Regulation of SAN by the Autonomic Nervous System and the Coupled-Clock System

Under physiological conditions, HRV is regulated by two main signaling cascades: the autonomic nervous system (ANS) and the coupled-clock system within the SANCs (27). The involvement of the ANS implicates the brain as a modulator of the SANCs automaticity through a balanced control of G-protein-coupled receptors (GPCRs) from both ANS branches: the sympathetic and the parasympathetic nervous system (SNS and PNS). Stimulation of the sympathetic nervous system (SNS) increases HR while stimulation of the parasympathetic nervous system (PNS) decreases the HR (28, 29). Acetylcholine that is released upon PNS stimulation acts on muscarinic receptors (M2R) in the SAN of the human heart and reduces its rate of firing (29).

The sympathetic activation of the heart (adrenergic), *via* the release of its neurotransmitter norepinephrine (NE), leads to the activation of the β-adrenergic receptors (β-AR) expressed at the sarcolemma of SANCs, thus causing the activation of the β-AR cyclase. The β-AR pathway involves the activation of adenylyl cyclase (AC), *via* the stimulatory guanosine triphosphate (GTP) regulatory protein (Gs), which converts adenosine triphosphate (ATP) into adenosine 3′, 5′- monophosphate (cAMP), which in turn stimulates cAMP-dependent protein kinase A (PKA) (30). PKA is a central mediator of β-AR regulation of cardiac function. Subsequently, a multitude of target proteins including the ryanodine receptors (RyR) and the sarco-/endoplasmic reticulum Ca^2+^ ATPase (SERCA) Ca^2+^ pumps in the SAN are phosphorylated by PKA to evoke intracellular Ca^2+^ [(Ca^2+^)_i_] oscillations affecting currents through the Na^+/^Ca^2+^ exchanger (NCX) (31). This surge of [Ca^2+^]_i_ ultimately increases the slope of the spontaneous diastolic depolarization (SDD) and consequently the heart rate (HR) (positive chronotropic effect) (32). There is increasing evidence that SAN pacemaker activity is subject to an intrinsic regulation by CaMKII *via* modulation of L-type Ca^2+^ currents (*I_Ca,L)_* inactivation and reactivation (31, 33). Additionally, the hyperpolarization-activated cyclic nucleotide-gated (HCN) channel gene family is a key determinant of mechanisms underlying chronotropic effects of β-AR stimulation (34). HCN4 channels are the most prevalent in SAN. These channels are regulated by PKA and therefore they are involved in the auto-pacing activity of SAN, as well as in the autonomic modulation of the HR (35).

In contrast, the parasympathetic input represses the rhythm of SANCs through the release of acetylcholine (ACh) which reduces the production of cAMP in SANCs. More importantly, ACh activates the potassium channels driving the SANCs into the hyperpolarized state. This leads to a reduced slope of SDD and a decrease in HR (negative chronotropic effect) (36). Nonetheless, exposure of SAN cells to adrenaline or acetylcholine revealed a shift in the pacemaker locus across the SAN tissues, thus implying a different expression pattern of the effectors/signaling cascades (to these neurotransmitters) by SANCs and reinstating their heterogeneity (18).

Although debatable, the “coupled-clock” system is believed to regulate the automaticity of the SANCs (7). It comprises an ensemble of highly dynamic membranes: the surface membrane (Sarcolemma-SL) and the sarcoplasmic reticulum (SR) membrane. The SL and SR membranes express ion channels and transporters defining the membrane clock or “M clock” and “Ca^2+^ clock”. Both clocks work interdependently but synergistically contribute to SDD, triggering the AP upstroke (37). The degree of the coupling between the “M” and the “Ca^2+^” clocks delineates the normal pacemaker function (7).

Electrophysiological studies identified electrical currents that form the pacemaker AP in SANCs. The ionic currents defining the “M clock” include the funny current (*I_f_*) carried by HCN channel (38), T-type (Cav3.1) calcium current (*I_Ca,T_*) (39), L-type (Cav1.3 and Cav1.2) calcium currents (*I_Ca,L_*) (40), sodium-calcium exchange current (*I_NCX_*) (41), and rapid and slow delayed rectifier potassium currents (*I_K,r_* and *I_K,s_*) (42, 43). Proteins defining the “Ca^2+^ clock” include RyR_2_ (44) and SERCA (45).

In general, local diastolic Ca^2+^ releases (LCRs) from the SR occur rhythmically. This promotes the Na^+^/Ca^2+^ exchange (NCX) current (*I*_NCX_) to generate a local AP which subsequently spreads as a depolarization wave over the SANCs and the neighboring myocardium (46). It is believed that the Ca^2+^-cAMP-PKA pathway is also involved in regulating the clock coupling (45). In fact, Ca^2+^ release stimulates both the CaMKII and PKA *via* Ca^2+^-calmodulin activated ACs. These kinases phosphorylate the SL “M clock” proteins and the SR Ca^2+^ cycling proteins, which promotes SR Ca^2+^ release, thus further promoting the Ca^2+^ -calmodulin-activated ACs and CaMKII (32). It has been shown that withdrawal of β-AR stimulation uncouples the clocks, thus failing to generate a spontaneous AP in SANCs (45). Recently, Sirenko and colleagues reported that the inhibition of the phosphoprotein phosphatases (PP) increases the firing rate of APs *via* the coupled-clock mechanism, including respective increases in the SR Ca^2+^ pumping rate, *I_Ca,L_*, and *I_NCX_* (46).

## 4 SAN Remodeling and Dysfunction in Diabetes

Diabetes mellitus (DM) is a global health problem that affects hundreds of millions of people worldwide (47). The chronic hyperglycemia seen in diabetes is precipitated due to abnormalities in insulin secretion, insulin action, or a combination of both in the form of insulin resistance. The prevalence worldwide ranges from around 5% to more than 15%. In the Middle East, the prevalence is reported to be among the highest in the world, with an average of 11.4% (48). The prevalence of cardiovascular risk factors is high among patients with diabetes and in those with earlier onset of the disease, where higher cardiovascular risks and poorer cardiovascular outcomes and mortality are seen (49). In fact, the leading cause of mortality among patients with diabetes is cardiovascular disease (50). Besides mechanical changes, alteration in the electrical function is another main characteristic of a diabetic heart.

Overall, metabolic abnormalities have been linked to a reduction in sympathetic activity and atypical SAN function (51, 52). This is mainly blamed on nerve growth factor production by adipocytes (53). Such release of growth factors can also account for the higher level of innervation seen in the nodal tissue (53). For example, the adipocyte-derived metabolic hormone leptin has also been suggested to be linked to the SAN function. Leptin receptor-deficient mice are at higher risk of developing arrhythmias due to a reduction in SAN recovery time and relative autonomic denervation (54). The SAN function has also been linked to metabolic changes through the role of free fatty acids (FFA) whereby the *I_f_* current is upregulated by FFA and sympathetic innervation, which results in increasing intracellular Ca^2+^ level and *I_st_* current (55, 56). Collectively, these studies demonstrate that diabetes induces metabolism-mediated dysfunction of the SAN.

### 4.1 Effects of the Impaired Autonomic Nervous System on SAN Function in Diabetic Conditions

One of the most important determinants of cardiac function and performance is the HR, which is modulated by the intrinsic rhythmical firing of the SAN. DM is associated with significant cardiovascular complications and neuropathies (57, 58). The regulation of HR by the ANS has been shown to be impaired in diabetic patients (59, 60). This condition is referred to as cardiovascular autonomic neuropathy (CAN), one of the earliest manifestations of DM (59). Up to 90% of diabetic patients exhibit CAN, which is often accompanied by impairment of the nerves of the heart and possible damage to the PNS mechanisms regulating HR (59, 60). These findings are of crucial clinical significance since DM patients with CAN have increased mortality as compared to patients with DM who do not exhibit CAN (61).

In type 2 DM (T2DM), PNS activity is impaired (59, 62). In addition, in type 1 DM (T1DM), there is an impairment in the mechanisms regulating HR by PNS, and that this impairment is accompanied by a change in the response of the SAN to PNS agonists such as carbachol (CCh) (63, 64). The impaired PNS activity and CAN are demonstrated earlier in T2DM as compared to T1DM (59); however, the mechanisms that are involved in the impaired regulation of HR by PNS in T2DM are not completely understood. It appears that the cardiac autonomic dysfunction that is associated with T2DM is mainly caused by direct damage to the autonomic nerves themselves (59, 65, 66); however, a direct impairment of the SAN function with T2DM cannot be ruled out at this time.

It is important to note that screening diabetic patients for cardiac autonomic neuropathy is highly recommended, especially in those patients with a history of macrovascular or microvascular complications, increased cardiovascular risk, and poor glycemic control (67). While cardiovascular reflex tests are still standard of care, the measurement of HRV is one of the most convenient, pain-free ways to reliably assess the cardiac autonomic neuropathy (68, 69). HRV is defined as the variation between two consecutive heartbeats. A decrease in ANS function has been reported to precede the progression into hypertension (70). Higher HRV indicates higher parasympathetic activity and reflects better adaptations to microenvironmental changes (71), and low HRV is reported as a marker of increased cardiovascular risk (69, 72). Among the general population, increased systolic blood pressure, abdominal diameter, waist–hip ratio, and body mass index (BMI) have been associated with decreased HRV (73, 74). HRV was reported to be improved following weight loss (75). No substantial data is available on the relationship of these parameters in the diabetic state, and the present data on HRV among patients with diabetes is contradictory (76, 77). Nevertheless, it has been reported that HRV is reduced in T1DM youth patients (78).

### 4.2 Cellular and Molecular Changes Within the Diabetic SAN Tissue

Irrespective of the modality of diabetes (T1DM vs. T2DM), manifestations of the sympathetic and parasympathetic axes certainly precipitate variabilities in cardiac rhythm. Despite this direct modulation of the SAN by the ANS, the cellular, molecular, and structural changes within the SAN tissue in hyperglycemic conditions remain ill-defined.

Spontaneous and rhythmical APs are generated by the SANCs, the primary pacemaker of the heart. Under normal circumstances, these spontaneous APs, and the slow diastolic depolarization between successive APs, contribute to the determination of the intrinsic HR (29). The length of time between successive APs in the myocytes of the SAN is determined by the contributions of ionic currents that can affect the transition from the maximum diastolic potential (MDP) to the threshold and the initiation of the next AP. The ionic currents include the hyperpolarization-activated current or funny current (*I_f_*), and a rapidly activating delayed rectifier K^+^ currents (*I_Kr_*) generated by ether-a-go-go (ERG) channels (7, 28, 79). The PNS reduces HR by activation of inhibitory G proteins associated with M2R and attenuating spontaneous firing of AP in SAN myocytes (58). While the primary pacemaker activity of the SAN is spontaneously generated, the rate of this activity and, therefore the HR, can be modified by factors such as ANS, hormones and neurotransmitters, medications, ions, hypoxia, as well as disease states.

In a recent study, Liu et al. (58) examined the effects of carbachol (CCh) on HR and SAN function in isolated SAN cardiomyocytes of male and female db/db mice, an animal model exhibiting features of T2DM. CCh showed an attenuated effect on slowing the spontaneous AP firing which was associated with a smaller decrease in the slope of diastolic depolarization and a reduced hyperpolarization of the MDP. CCh did not produce significant hyperpolarization in SAN cardiomyocytes isolated from male and female db/db mice (58). The results of this study provided some evidence for cellular and molecular mechanisms that might lead to the attenuated regulation of PNS on HR in T2DM (58). The acetylcholine-activated K^+^ current (*I_KACh_*) becomes desensitized and its amplitude diminishes in the presence of an M2R agonist such as CCh (80–82). This channel, *I_KACh_*, which mediates the activation of PNS in the SAN, was found to exhibit increased desensitization and faster deactivation kinetics in the SAN of db/db mice, resulting in an attenuated effect of CCh on HR in these mice. Furthermore, the impaired *I_KACh_* in SAN myocytes was attributed to altered G protein signaling 4 (RGS4) and phosphatidylinositol (3,4,5) P3 (PIP3) signaling. The authors concluded that their findings may identify new interventions that might benefit diabetic patients with CAN, and with attenuated ANS signaling to the SAN (58). In support of this contention, earlier studies have also shown that the kinetics of *I_KACh_* are regulated by RGS4 and PIP3 signaling in the SAN (83). RGS4 is inhibited by PIP3 which is activated by insulin-mediated phosphoinositide-3 kinase (PI3K) signaling. Therefore, impaired insulin and PI3K signaling in T2DM could result in enhanced RGS4 activity due to a loss of PIP3-mediated inhibition of RGS4 (84–86).

At the molecular level, the CaMKII pathway, known to maintain the pacemaker activity of SAN (33) is altered in diabetes and could lead to SAN anomalies. This is mainly achieved through the oxidation of CaMKII into ox-CaMKII which slows down the pacemaker activity of the SAN and promotes apoptosis of SANCs leading to high mortality in human and in experimentally diabetic animal models (33, 87, 88). In fact, inhibition of CaMKII oxidation (mice lacking active NADPH oxidase), or pharmacological inhibition of CaMKII concedes resistance to apoptosis and fibrosis for injured SAN (87). Furthermore, using antioxidants in streptozotocin (STZ)-treated rats successfully reversed this process (87–89).

In addition to CaMKII sustained activity, hyperglycemia furthers the production of detrimental ROS that damage the cells and induce apoptosis leading to reduced SAN function. High blood glucose levels in diabetes increases diacylglycerol (DAG) content that activates protein kinase C (PKC). This in turn phosphorylates NADPH-oxidase, leading to further production of ROS. Such inflammatory reactions may also be associated with the action of advanced glycation end products (AGEs), which interact with their respective receptors (RAGE) on the cell surfaces, to produce the proinflammatory cytokines. Collectively, these promote inflammation, oxidative stress, and cellular death (90).

In SAN, basal PKC activity is crucial for normal spontaneous SANC activity. In freshly isolated rabbit SANCs, inhibition of PKC suppressed SR Ca^2+^ cycling and the spontaneous beating of the cells. Normally, SANCs firing is regulated by local subsarcolemmal Ca^2+^ release from the SR. Such release takes place during the SDD and stimulates NCX leading to an increased rate of SDD and acceleration of spontaneous SANC activity (91).

**Figure 1:**
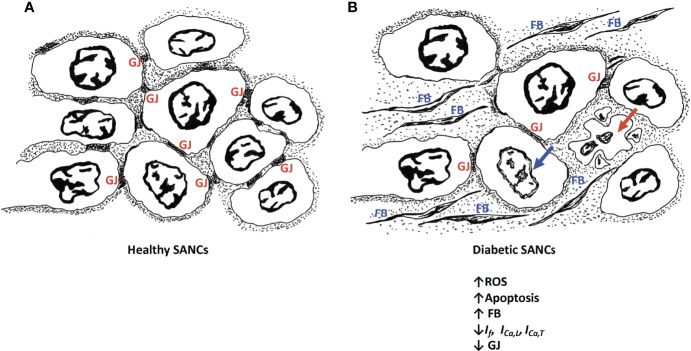
Schematic diagram of healthy SANCs showing intact gap junctions (GJ) **(A)** and remodeling of the diabetic SANCs showing decreased GAP junctions, infiltration of fibrous tissues, and apoptosis of SANCs **(B)**. Red arrow represents cell apoptosis; blue arrow represents nuclear blebbing; GJs are characterized by altered expression of connexins. Fibroblasts (FB) are populating in the extracellular matrix.

The pathological remodeling of the SAN in diabetic conditions resembles the one noticed in the elderly, whereby nodal cells are atrophied and are surrounded by infiltrated fibrous tissues (25, 26) (**Figure 1**). This profoundly alters the electrical conduction between the SANCs. In fact, the diabetes-induced fibrosis in STZ-treated rats detrimentally reduces the conduction properties of SAN tissues, often depicted by a wider P wave on the ECG tracings (92, 93). In contrast, the remodeling of the SAN in obese rats is manifested by hypertrophied SANCs and enlarged SAN tissues (94). Moreover, in diabetic conditions, the adenosine receptor AR1 is upregulated in the heart (95). Functional analysis showed that the upregulation of AR1 promotes SAN dysfunction and nodal conduction abnormalities (96). Thus, the direct effect of diabetes on SAN safety cannot be simply ignored. In addition, the indirect effect of diabetes on SAN tissues exemplified by the hypoperfusion of the SAN by the coronary arteries, due to their pathological remodeling in hyperglycemic conditions, profoundly alters SAN function.

### 4.3 Insights on Ion Channels Dysfunction and Cytoskeletal Remodeling in Diabetic SAN

Disruption of cardiac electrical activity has been widely observed in the hearts of both T1DM and T2DM patients (97). For example, prolongation of QRS and QT segments (98), disturbance in automaticity of SAN, atrioventricular block, and left bundle branch block have been commonly reported (99–101). T2DM patients have a high risk of atrial fibrillation (102, 103), ventricular arrhythmia (104), and fibrillation (105).

Similarly, prolonged QRS and QT intervals have also been reported in animal studies on diabetes. In isolated perfused hearts of non-obese Goto-Kakizaki (GK) T2DM rats, spontaneous HR was lower compared to control rats. This observation implies that the changes are, at least in part, due to an intrinsic abnormality in the cardiac electrical conduction system (106). Although earlier studies in animal models have reported a profound decrease in HR and increased mortality in diabetic animals (106), reports on SAN remodeling in diabetes are limited. In STZ-treated diabetic rats, HR was found to be slower compared to controls. Furthermore, SAN conduction and pacemaker cycle length and time were prolonged (107). Recently, in a recent, in leptin-receptor deficient T2DM diabetic mice, disturbance of systolic and diastolic activity of the heart, prolongation of SAN recovery time and relative autonomic denervation were reported (54).

**Figure 2:**
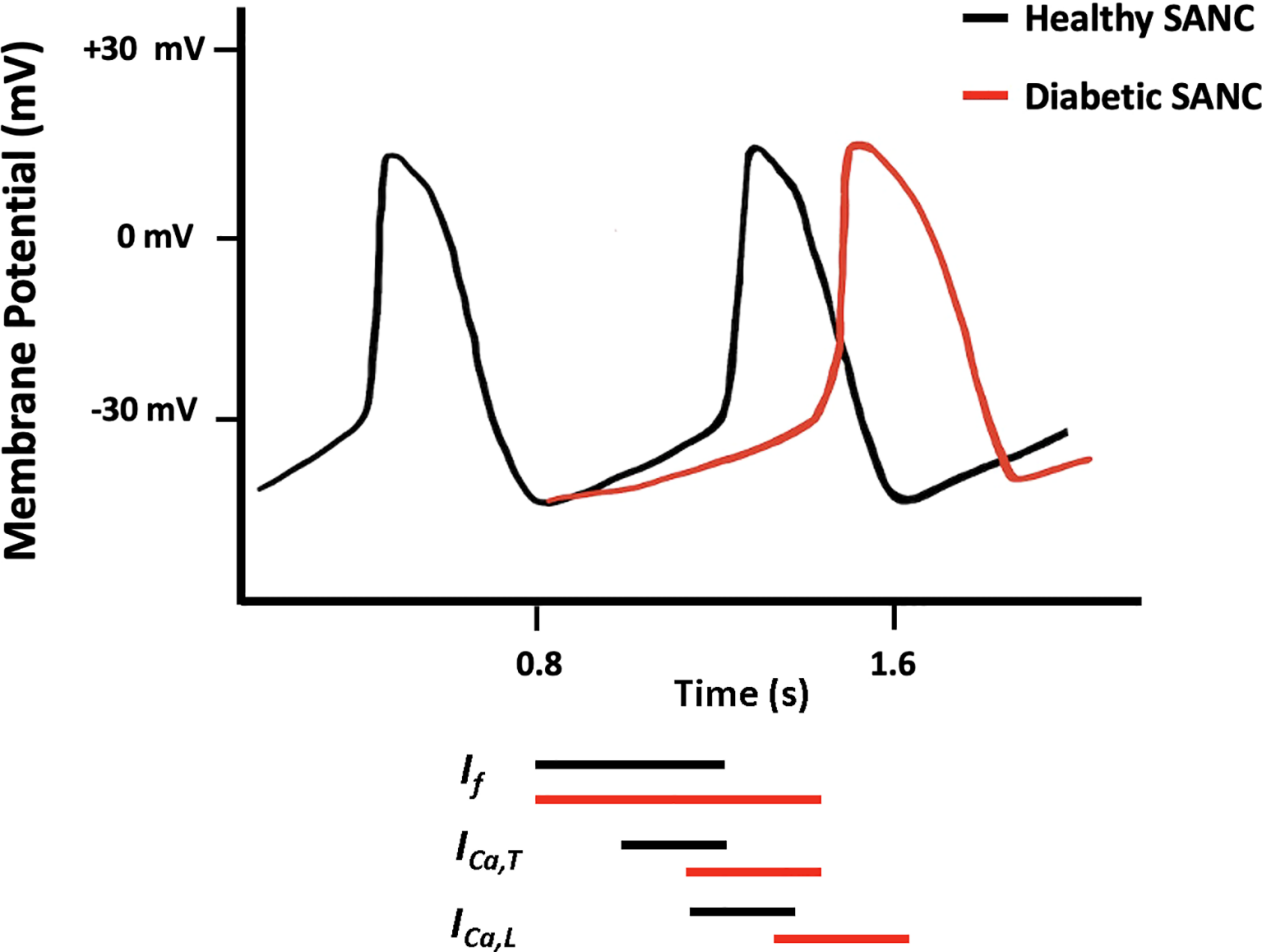
Schematic diagram showing action potential (AP) pattern of SANCs in control (black) and diabetic (red) SAN.

Structural and/or functional channelopathies are thought to play a role in the electrical abnormalities reported in the diabetic heart. At the cellular level, diabetes reduces the velocity of SAN conduction and the slope of SDD and prolongs the duration of cardiac AP which is attributed to the altered expression and electrophysiological properties of various ion channels (97, 108–110) (**Figure 2**).

#### 4.3.1 SAN Ion Channels

**Figure 3:**
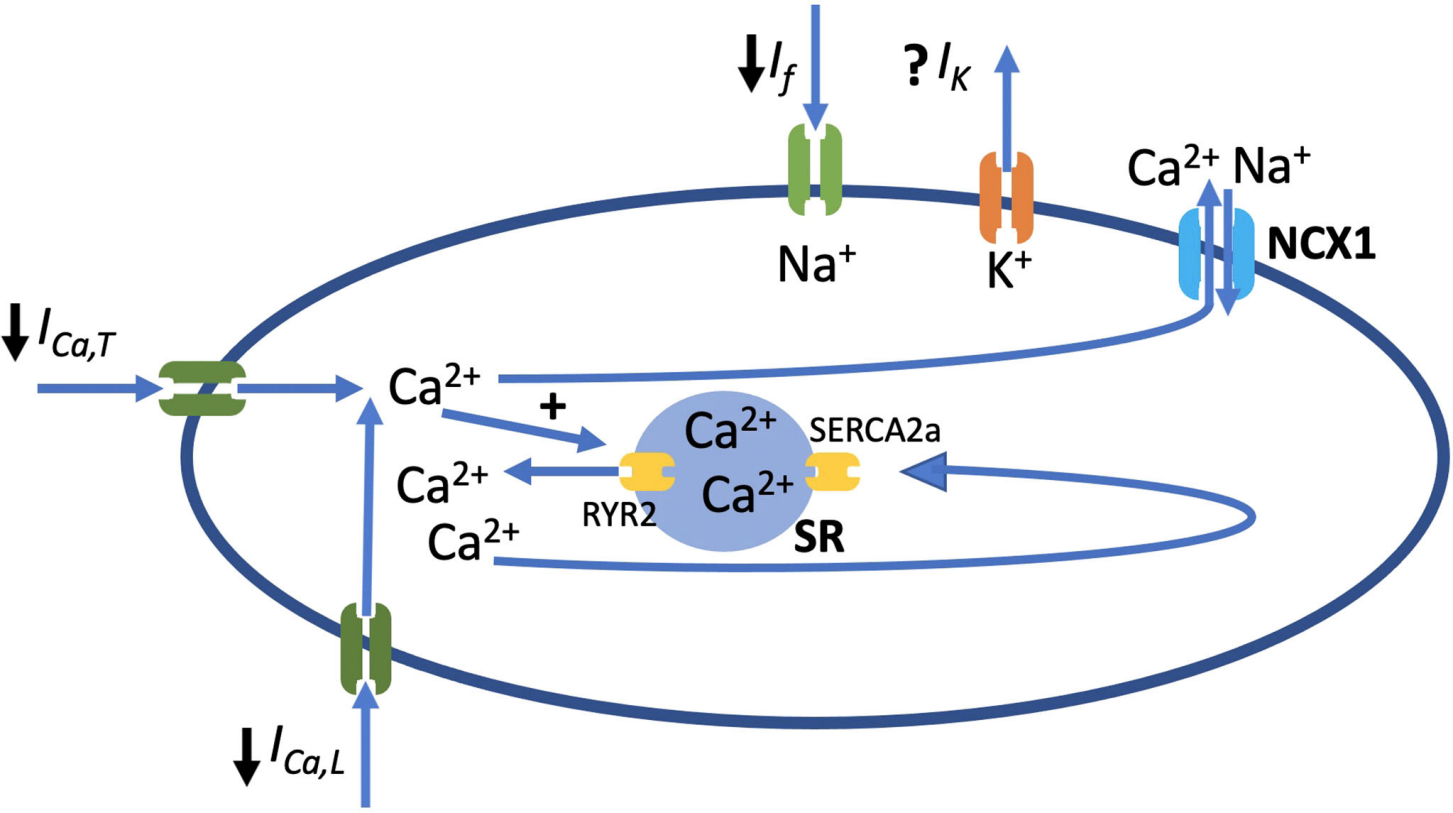
Schematic diagram of SANC showing main ion channels that play a role in AP generation and along with ryanodine receptor 2 (RyR2), the sarcoplasmic reticulum (SR) calcium pump (SERCA2a) and the Na^+^/Ca^2+^ exchanger (NCX1). ↓ represents the currents that play a main role in generation of AP and are inhibited in diabetes. *I_f_*, funny current; *I_Ca,T_*, current conducted by T-type voltage-gated Ca^2+^ channel; *I_Ca,L_*, current conducted by L-type voltage-gated Ca^2+^ channel.

As mentioned earlier, the pacemaker activity of the SAN is regulated by several ion channels that are thought to contribute to the SDD. Specifically, the pacemaker activity is determined by a net inward current caused by the deactivation of outward current (*I_K,s_*) and the activation of inward currents, carried mainly by voltage-gated sodium channels (Nav1.5), hyperpolarization-activated cyclic nucleotide-gated channels (HCN), Cav3.1 channels and Cav1.3 channels (1, 12, 111). Therefore, disruption of one or more of these ion channels would alter the electrophysiological properties of the SAN. **Figure 3** shows the main ionic currents involved in the AP of SANCs.

##### 4.3.1.1 Voltage Gated Sodium Channel (Nav1.5)

The voltage-gated sodium channel (Nav1.5) is predominantly expressed in the heart and governs the upstroke of the AP in the atrial and ventricular cells (112). However, its expression and distribution in the SAN tissues remain debatable. While studies showed the presence of the Nav1.5 in SAN tissues, others indicated that they are mainly expressed at the periphery but absent from the center of the SAN (113). Moreover, developmental studies showed that this particular isoform of the sodium channel is highly expressed at an early stage during development, and its expression is reduced later on when the heart adopts a slower rate of contraction (114). From the functional point of view, while studies showed an implication of Nav1.5 in the conduction of SANCs (115), others indicated that Nav1.5 rescues the pacing activity of SANs in hyperpolarization conditions (114).

In heterozygous knockout mice Nav1.5^+/−^, delayed conduction and arrhythmia were reported (116). In alloxan-induced diabetic rabbits, the protein level of Nav1.5 and the density of *I_Na_* in the ventricle were significantly reduced (117). In agreement with this finding, Zhang et al. found that Nav1.5 was significantly decreased in the left atrium and right ventricle by 33% and 37%, respectively (118). However, Ferdous, et al. (2016) showed an upregulation of the gene encoding Nav1.5 in the SAN of STZ diabetic heart compared to normal (107). Such change in the expression of Nav1.5 could be an important determinant for arrhythmogenesis observed in diabetes. Furthermore, increased production of ROS in diabetic hearts, which is known to modify the properties of Na^+^ channels, could also contribute to the impaired SAN function in diabetes (119, 120).

##### 4.3.1.2 Hyperpolarization-Activated Cyclic Nucleotide-Gated Channel

HCN channel conducts the hyperpolarization current (funny current; *I_f_*). The channel activity is responsible for the spontaneous diastolic membrane depolarization of the SANCs and therefore the generation of spontaneous SAN AP. The channel is a tetramer that is composed of four HCN subunits that are made of six transmembrane segments. Although four HCN gene family members (HCN1, HCN2, HCN3 and HCN4) have been found in the heart, HCN4 is the prominent HCN in human SAN (121).

The HCN channel is modulated by cAMP (122). Specifically, the activity of the channel rises with the increase in intracellular concentrations of cAMP, which binds to the C-terminus of the channel (123). While cAMP activates the channel, muscarinic agonists inhibit the inward current at diastolic potentials by shifting the activation curve to more negative values (124). It is believed that regulation of HCN4 by cAMP in the heart is essential for HR modulation by the ANS. While cAMP-dependent increase in HCN4 activity is needed for the acceleration of the HR under ANS stimulation, a drop in the levels of cAMP following vagal stimulation lessens HCN4 activity and decreases HR (28, 123).

Studies in animal models have reported that SANCs from knockout mice lacking HCN4 have a parallel decrease of 75% in *I_f_* (125). In STZ T1DM, altered mRNA expression of HCN4 channels was reported (107). In support of this finding, a recent study conducted by Zhang et al. (2019) has shown a significant disturbance in the ECG characteristics of STZ-treated diabetic rats. This change was marked by prolongation of the PR interval, RR interval, QT interval and QRS complex. In addition, the beating rate of SAN was slower compared to control. Electrophysiological experiments showed that blocking of HCN4 channels with CsCl resulted in reduction of the beating rate of rat SAN preparations, indicating that HCN4 channels play a vital role in sustaining the spontaneous pacemaker activity. Along with the downregulation of HCN4 in the SAN, *I_f_* was significantly reduced which could explain the observed reduction in SAN function (118).

Similarly, altered expression of HCN4 was reported in T2DM animals. *In vivo* experiments conducted in GK diabetic rats have shown that a decrease in the HR of young animals was coupled to downregulation of the expression of HCN4 gene (126). It is important to note that downregulation of HCN4 and *I*_f_ might be partly responsible for bradyarrhythmia observed in diabetic patients (127).

Although elevated ROS is a consistent finding in cardiovascular complications of diabetes, data on defined molecular targets and pathways that connect increased oxidation with SAN function are very limited. In sick sinus syndrome, mitochondrial oxidative stress was found to induce HCN4 downregulation (128). A recent study found that this effect involves the inhibition of mitochondrial thioredoxin-2, an important ROS scavenger that regulates the mitochondrial apoptosis signaling pathway. Indeed, the deletion of thioredoxin-2 in the whole mouse heart caused dilated cardiomyopathy, atrioventricular block and sinus bradycardia. The mice also displayed reduced expression of HCN4 in SANCs and typical electrophysiological signs of sick sinus syndrome (129).

##### 4.3.1.3 T-Type Voltage-Gated Ca^2+^ Channel (Cav3.1)

The T-type voltage-gated Ca^2+^ channel plays a key role in cell excitation and Ca^2+^ handling in the heart. Three types of T-type Ca^2+^ channels were cloned; Cav3.1 (α1G), Ca_v_3.2 (α1H) and Ca_v_3.3 (α1I) (130). The Cav3.1 is highly expressed in the SAN and atrioventricular node, while it is poorly expressed in the myocardium. Cav3.1 contributes to the total Ca^2+^ current in the SAN. It was found to play a vital role in pace-making and impulse conduction in both mice and humans (131). The current generated by Cav3.1(*I_Ca,T_)* is marked by delayed activation at voltages expanding over the diastolic depolarization phase, and rapid inactivation (132). It was reported that mice deficient in Cav3.1 had slower SAN recovery time, decelerated pacemaker activity of SAN and HR, and delayed atrioventricular conduction. These findings show that Cav3.1 is a major contributor to the generation of cardiac rhythmicity (132). An earlier study demonstrated that *I_Ca,T_* of mouse SAN is activated by isoproterenol in a PKA-dependent manner; however, the physiological role of adrenergic control of *I_Ca,T_* is not well-understood (130).

Studies on the expression of Cav3.1 in diabetic animals reported conflicting results. Ferdous et al. (2016) have shown that in the SAN of STZ diabetic rats, mRNA expression of Cav3.1 is upregulated (107). In agreement with this finding, earlier studies have reported upregulation of Cav3.1 in the ventricle from the GK and the Zucker diabetic fatty rat (133, 134). It is expected that the upregulation of Cav3.1 upsurges T-type Ca^2+^ current and, consequently the slope of the pacemaker potential and HR in STZ rats. In contrast and in a more recent study, it has been shown that the expression of Cav3.1 gene is downregulated by three folds in GK rats. As a result, reductions in pacemaker activity and the slope of diastolic depolarization are expected (126, 132).

##### 4.3.1.4 L-Type Voltage-Gated Ca^2+^ Channel (Cav1.3)

Two types of L-type voltage-gated Ca^2+^ channels, namely Cav1.2 and Cav1.3 are expressed in the heart; however, Cav1.3 is predominantly expressed in the SAN and atrioventricular node. In SAN, Cav1.3 not only contributes to the pacemaker activity but also to the initiation of diastolic depolarization and regulation of Ca^2+^ release from SR during SAN pacemaker activity (135, 136). Evidence shows that downregulation of the Cav1.3 gene in mice causes a reduction in pacemaker activity and causes spontaneous arrhythmia in SANCs (135).

Experiments in isolated perfused hearts of STZ diabetic animals have shown that decreased HR and prolonged SAN AP were associated with a significant decrease (32%) in the expression of Cav1.3 gene in diabetic SAN compared to control SAN (118). Previous studies have shown that deletion of the channel (Cav1.3^–/–^) caused the prolongation of the PR interval and the complete block of the atrioventricular conduction (137). As a result, downregulation of Cav1.3 could be a source of bradyarrhythmia and heart block in patients with T1DM. In GK diabetic rats, SAN Cav1.3 was also downregulated (126). This reduction in *I_Ca,T_* observed in diabetes may be attributed to the change in the activation/inactivation kinetics of the channels, the expression of the channel proteins, or the change in the single-channel conductance. Although several studies have investigated the effect of diabetes on such electrophysiological properties in ventricular myocytes, the data on SAN Cav1.3 is scarce (138).

An earlier study examined the effect of CaMKII on SAN spontaneous excitation and modulation of *I_Ca,L_* in freshly isolated rabbit single SANCs. It was found that inhibition of CaMKII can completely arrest SANCs largely as a result of depressed *I_Ca, L_* amplitude, reduced window current, and slowed recovery of L-type Ca^2+^ channels from inactivation. This finding shows a key role of CaMKII in regulating cardiac pacemaker activity (33).

#### 4.3.2 SAN Cytoskeletal Proteins

Cytoskeleton proteins support cell shape, elasticity, and contractility. For example, collagen I is an extracellular protein that provides a structural framework to the cardiac myocytes, stiffness to the myocardial wall, and assists in force transmission (139). Therefore, changes in the level of expression of this protein affects the conduction properties in the heart, thus promoting arrhythmogenesis. In fact, several studies have shown that collagen I is overexpressed in diabetic rat hearts, which would contribute to the decreased ventricular compliance (140). A recent study in STZ diabetic rats has shown an increase in the level of collagen I expression throughout the conduction system in the heart including the SAN (118).

Caveolin-3 is a structural and regulatory protein that modulates the function of ion channels, including those involved in pace-making (141). Mutations in the gene encoding caveolin-3 have been linked to several cardiac diseases such as long QT syndrome, myocardial hypertrophy, and diabetic cardiomyopathy (142, 143). SAN cells are rich in caveolin-3 protein (144). Studies have shown that caveolin-3 co-localizes with HCN4 channel and affects its function (145). The expression of caveolin-3 mutant modifies the gating properties of HCN4 channels by causing a rightward shift in the activation curve (146). Furthermore, when caveolae are disturbed in the SAN, β2-adrenergic receptor regulation of HCN4 channel was found to be lost (145). In spontaneously beating neonatal cardiac cells, the T78M mutation of the caveolin-3 gene significantly enhanced peak-to-peak AP variability linked to rightward shift of activation potential of HCN4 channels (147). In support of the vital role of Caveolin-3 in SAN function, Lang et al. (2016) found that in caveolin-3 knockout mice, heart rate fluctuation with altering periods of bradycardia-tachycardia rhythm was observed. Such change was linked to the disturbance of SAN function (144). In chronic STZ-induced diabetic rats, lower expression of caveolin-3 was detected (148, 149).

PKC-β2 is known to be overexpressed in the diabetic heart and contributes to cardiomyocyte hypertrophy in diabetes. Interestingly, impairment in caveolin-3 expression was found to be one of the underlying mechanisms for cardiac dysfunction that is linked to hyperglycemia-induced PKC-β2 activation (150). Therefore, it is expected that changes in the expression of caveolin-3 in any region in the heart may contribute to the pathogenesis of diabetic cardiomyopathy. However, data on the altered expression of caveolin-3 in the SAN of diabetic rats is scarce. In addition, α-actinin is another structural protein involved in maintaining cell shape and contractility. A significant decrease in the expression of α-actinin was observed throughout the heart regions but not in the diabetic SAN (118).

A hallmark of cell-to-cell coupling is the gap junction at the intercalated discs, forming macrochannels for effective transmission and propagation of the AP between cardiac cells. The expression and distribution of the different variants of connexins-Cx (the proteins forming gap junctions) is specific to each different area of the SAN (151). While Cx40, Cx45, and Cx46 are abundantly distributed in the central area of the SAN, Cx43 is almost non-existent in this tissue (12). The heterogeneity of the expression of these Gap junction proteins explains the distinctiveness of conduction through the SAN. Of interest, one of the striking remodeling features in diabetic cardiomyopathy is the persistently reduced transmission of the AP through gap junctions. In fact, the function of Cx43, which is predominantly expressed in the working myocardium, depends on tyrosine phosphorylation. Both the expression/distribution and phosphorylation of Cx43 are altered in diabetic hearts, leading to reduced impulse propagation which increases the risk for the development of fibrillation (152, 153). In SAN from diabetic animal models, the expression of Cx40, Cx43, and Cx45 is still controversial. Howarth et al. (2007) showed a moderate increase of these connexins in the SAN of the STZ-treated rats (154). However, Ferdous et al. (2016) reported an increase in the mRNA of Cx45 in the SAN of STZ-treated rats, without a significant change in the expression pattern of the Cx40 and Cx43 (107). The literature falls short on the implication of Cx40 and Cx45 in the electrical remodeling of the diabetic SAN, the slow conductance of the AP, and consequently cardiac rhythm. This warrants extensive investigation to fill this gap in the literature and to help stabilize cardiac rhythm in diabetic conditions.

### 4.4 Electrophysiological Effects of Anti-Diabetic Drugs on SAN Currents

While few studies indicate that anti-diabetic drugs significantly protect the cardiovascular system, other controversial reports conclude that anti-diabetic medicine is far from reversing the cardiac complications manifested in diabetic settings (155). For example, Metformin, a medicine used to treat T2DM, stimulates the 5′ adenosine monophosphate-activated protein kinase (AMPK) signaling pathway, which consequently stimulates the ATP-sensitive potassium channels (K_ATP_) involved in the control of HR (156). In the same vein, the activation of AMPK contributes to the maintenance of the *I_Ca,L_*, the *I_Ca,L_*- triggered Ca^2+^ transients amplitude, the Ca^2+^ content, and promotes cell contraction (157).

Rosiglitazone, the thiazolidinedione class of anti-diabetic drugs, acts to inhibit K_ATP_ channels in the pancreatic β-cells (158). Inhibition of these channels by high levels of ATP causes membrane depolarization, Ca^2+^ influx through voltage-gated Ca^2+^ channels and Ca^2+^ -dependent secretion of insulin; thus inhibition of pancreatic K_ATP_ channels prevents insulin secretion (159). Conversely, the blockade of the cardiac isoform of the K_ATP_ channel (K_IR_6.2/SUR2A) severely compromises the cardiac ability to cope with ischemic assaults (158, 160–162), implying that the K_ATP_ of SANCs would be tremendously altered in diabetic patients on Rosiglitazone treatment. However, existing studies do not provide confirmatory information on the role of anti-diabetic drugs on the AP of SANCs. This field warrants further investigations.

While insulin remains an ultimate treatment for a large group of diabetic patients, its effect on the electrophysiological properties of cardiac cells remains inexplicit. However, few studies highlighted an implication of insulin in the electrical activity of cardiac myocytes. For example, in myocytes isolated from T1DM diabetic hearts, the main affected repolarizing current is the *I_to_* (138). In an earlier report (1999), Shimoni and colleagues have shown that insulin treatment of isolated ventricular myocytes from STZ diabetic rats reverses the depression of ventricular K^+^ currents (163). Moreover, insulin mediates its effect through insulin receptors and the cAMP-dependent PKA to stimulate *I_Ca,L_* in isolated rat ventricular myocytes in a dose-dependent and reversible manner (164). Interestingly, insulin may also play a role in both the “M” and “Ca^2+^” clock systems by stimulating Ca^2+^-ATPase activity at the SR and the SL, and by increasing the Na^+^/Ca^2+^ exchange activity (164).

It is noticed that in insulin-resistant cardiac tissues, the normal rise in a calcium-independent sustained K^+^ current is either reduced or eradicated (165). The addition of insulin to myocytes isolated from insulin-resistant rats, treated with metformin (an insulin-sensitizing drug), produced a very significant enhancement of sustained K^+^ current (165). Thus, Metformin modulates the insulin resistance effect observed for cardiac K^+^ current which implies that these changes may also occur at the SAN level. In addition, both acute and chronic insulin treatment improved sodium currents in the atrial muscle of T1DM Atika mice by promoting the electrical properties of the Nav1.5 channel and by increasing its expression. This stabilization effect of insulin on atrial fibrillation could probably be stretched on SAN tissues since they express the Nav1.5 channel, and since arrhythmic SANCs are a hallmark of diabetes in animal models (54, 166).

## 5 Conclusion and Future Perspectives

Despite its primordial role in generating the AP waves that drive cardiac contractions, the dynamics of the SAN in diabetic settings remain ill-defined. It is clear that hyperglycemia modulates the expression/function of the ion channels underlying the electrical properties of the SANCs. This is manifested by repression of the currents involved in the upstroke of the AP and the conduction of SAN tissues. Chronic anti-diabetes treatments (metformin, insulin, rosiglitazone) adversely affect the SAN and, consequently, cardiac function. This is in addition to the structural/functional changes that underscore diabetic cardiomyopathy (working myocardium and other parts of the conduction system, i.e., the atrioventricular node and Purkinje fibers). Although various animal models are now established to study diabetes, the literature falls short on the synergy of the SAN during this metabolic syndrome. This includes a gap in the knowledge on the structural remodeling of SAN in diabetes as well as the metabolic changes induced by diabetes in the SANC. This calls for more investigations focused on the molecular signature of diabetes-induced SAN dysfunction to build an informative platform that links the clinical aspect of SAN failure with diabetes, and to provide a safer therapeutic approach for this metabolic disorder of the cardiac pacemaker. The advent of live imaging and optical mapping provides unique tools to study the metabolic and electrical changes in human diabetic SANCs. An alternative and more attractive approach would be a targeted therapy (pharmacological or genetic) of the SAN in diabetic subjects to warrant normal pacemaker activity and typical cardiac function. The different modalities of diabetic SAN injuries showcased herein must be carefully considered in the field of biological SAN engineering to develop a diabetes-resistant SAN for patients with diabetes.

## Author Contributions

LTA and MN: Conceptualization of the manuscript. LTA, SC, EA, AAK, MN: Writing the original manuscript and editing. All authors contributed to the article and approved the submitted version.

## Conflict of Interest

The authors declare that the research was conducted in the absence of any commercial or financial relationships that could be construed as a potential conflict of interest.

## Publisher’s Note

All claims expressed in this article are solely those of the authors and do not necessarily represent those of their affiliated organizations, or those of the publisher, the editors and the reviewers. Any product that may be evaluated in this article, or claim that may be made by its manufacturer, is not guaranteed or endorsed by the publisher.

## References

[1] Monfredi O Dobrzynski H Mondal T Boyett MR Morris GM. The Anatomy and Physiology of the Sinoatrial Node–a Contemporary Review. Pacing Clin Electrophysiol (2010) 33(11):1392–406. doi: 10.1111/j.1540-8159.2010.02838.x 20946278

[2] Dobrzynski H Boyett MR Anderson RH. New Insights into Pacemaker Activity: Promoting Understanding of Sick Sinus Syndrome. Circulation (2007) 115(14):1921–32. doi: 10.1161/CIRCULATIONAHA.106.616011 17420362

[3] Keith A Flack M. The Form and Nature of the Muscular Connections between the Primary Divisions of the Vertebrate Heart. J Anat Physiol (1907) 41(Pt 3):172–89. PMC1289112 17232727

[4] Sanchez-Quintana D Cabrera JA Farre J Climent V Anderson RH Ho SY. Sinus Node Revisited in the Era of Electroanatomical Mapping and Catheter Ablation. Heart (2005) 91(2):189–94. doi: 10.1136/hrt.2003.031542 PMC1768731 15657230

[5] Matsuyama TA Inoue S Kobayashi Y Sakai T Saito T Katagiri T. Anatomical Diversity and Age-Related Histological Changes in the Human Right Atrial Posterolateral Wall. Europace (2004) 6(4):307–15. doi: 10.1016/j.eupc.2004.03.011 15172655

[6] Efimov IR Fedorov VV Joung B Lin SF. Mapping Cardiac Pacemaker Circuits: Methodological Puzzles of the Sinoatrial Node Optical Mapping. Circ Res (2010) 106(2):255–71. doi: 10.1161/CIRCRESAHA.109.209841 PMC2818830 20133911

[7] Lakatta EG Maltsev VA. Vinogradova TM. A Coupled System of Intracellular Ca2+ Clocks and Surface Membrane Voltage Clocks Controls the Timekeeping Mechanism of the Heart's Pacemaker. Circ Res (2010) 106(4):659–73. doi: 10.1161/CIRCRESAHA.109.206078 PMC2837285 20203315

[8] Aziz Q Li Y Tinker A. Potassium Channels in the Sinoatrial Node and Their Role in Heart Rate Control. Channels (Austin) (2018) 12(1):356–66. doi: 10.1080/19336950.2018.1532255 PMC6207292 30301404

[9] Verkerk AO Wilders R van Borren MM Peters RJ Broekhuis E Lam K. Pacemaker Current (I(F)) in the Human Sinoatrial Node. Eur Heart J (2007) 28(20):2472–8. doi: 10.1093/eurheartj/ehm339 17823213

[10] Verheijck EE van Ginneken AC Wilders R Bouman LN. Contribution of L-Type Ca2+ Current to Electrical Activity in Sinoatrial Nodal Myocytes of Rabbits. Am J Physiol (1999). doi: 10.1152/ajpheart.1999.276.3.H1064 10070093

[11] Csepe TA Zhao J Hansen BJ Li N Sul LV Lim P. Human Sinoatrial Node Structure: 3d Microanatomy of Sinoatrial Conduction Pathways. Prog Biophys Mol Biol (2016) 120(1-3):164–78. doi: 10.1016/j.pbiomolbio.2015.12.011 PMC4808362 26743207

[12] Boyett MR Honjo H Kodama I. The Sinoatrial Node, a Heterogeneous Pacemaker Structure. Cardiovasc Res (2000) 47(4):658–87. doi: 10.1016/s0008-6363(00)00135-8 10974216

[13] Bartos DC Grandi E Ripplinger CM. Ion Channels in the Heart. Compr Physiol (2015) 5(3):1423–64. doi: 10.1002/cphy.c140069 PMC4516287 26140724

[14] Busquet J Fontan F Anderson RH Ho SY Davies MJ. The Surgical Significance of the Atrial Branches of the Coronary Arteries. Int J Cardiol (1984) 6(2):223–36. doi: 10.1016/0167-5273(84)90357-7 6469406

[15] Ramanathan L Shetty P Nayak SR Krishnamurthy A Chettiar GK Chockalingam A. Origin of the Sinoatrial and Atrioventricular Nodal Arteries in South Indians: An Angiographic Study. Arq Bras Cardiol (2009) 92(5):314–9. doi: 10.1590/s0066-782x2009000500002 19629284

[16] Saremi F Abolhoda A Ashikyan O Milliken JC Narula J Gurudevan SV. Arterial Supply to Sinuatrial and Atrioventricular Nodes: Imaging with Multidetector Ct. Radiology (2008) 246(1):99–107. doi: 10.1148/radiol.2461070030 18024438

[17] Zhou B. Sinoatrial Node Pacemaker Cells: Cardiomyocyte- or Neuron-Like Cells? Protein Cell (2021) 12(7):518–9. doi: 10.1007/s13238-021-00827-w PMC8225711 33550511

[18] Mackaay AJ Op't Hof T Bleeker WK Jongsma HJ Bouman LN. Interaction of Adrenaline and Acetylcholine on Cardiac Pacemaker Function. Functional Inhomogeneity of the Rabbit Sinus Node. J Pharmacol Exp Ther (1980) 214(2):417–22. 7391985

[19] Unudurthi SD Wolf RM Hund TJ. Role of Sinoatrial Node Architecture in Maintaining a Balanced Source-Sink Relationship and Synchronous Cardiac Pacemaking. Front Physiol (2014) 5:446. doi: 10.3389/fphys.2014.00446 25505419 PMC4244803

[20] Balbi T Ghimenton C Pasquinelli G Foroni L Grillini M Pierini G. Advancement in the Examination of the Human Cardiac Sinus Node: An Unexpected Architecture and a Novel Cell Type Could Interest the Forensic Science. Am J Forensic Med Pathol (2011) 32(2):112–8. doi: 10.1097/PAF.0b013e3181ce9f23 20679878

[21] Sano T Yamagishi S. Spread of Excitation from the Sinus Node. Circ Res (1965) 16:423–30. doi: 10.1161/01.res.16.5.423 14289151

[22] Christoffels VM Moorman AF. Development of the Cardiac Conduction System: Why Are Some Regions of the Heart More Arrhythmogenic Than Others? Circ Arrhythm Electrophysiol (2009) 2(2):195–207. doi: 10.1161/CIRCEP.108.829341 19808465

[23] Yi T Wong J Feller E Sink S Taghli-Lamallem O Wen J. Electrophysiological Mapping of Embryonic Mouse Hearts: Mechanisms for Developmental Pacemaker Switch and Internodal Conduction Pathway. J Cardiovasc Electrophysiol (2012) 23(3):309–18. doi: 10.1111/j.1540-8167.2011.02191.x PMC3749437 21985309

[24] Erokhina IL Rumyantsev PP. Ultrastructure of DNA-Synthesizing and Mitotically Dividing Myocytes in Sinoatrial Node of Mouse Embryonal Heart. J Mol Cell Cardiol (1986) 18(12):1219–31. doi: 10.1016/s0022-2828(86)80426-6 3820314

[25] Shiraishi I Takamatsu T Minamikawa T Onouchi Z Fujita S. Quantitative Histological Analysis of the Human Sinoatrial Node During Growth and Aging. Circulation (1992) 85(6):2176–84. doi: 10.1161/01.cir.85.6.2176 1591834

[26] Thery C Gosselin B Lekieffre J Warembourg H. Pathology of Sinoatrial Node. Correlations with Electrocardiographic Findings in 111 Patients. Am Heart J (1977) 93(6):735–40. doi: 10.1016/s0002-8703(77)80070-7 871100

[27] Rosenberg AA Weiser-Bitoun I Billman GE Yaniv Y. Signatures of the Autonomic Nervous System and the Heart's Pacemaker Cells in Canine Electrocardiograms and Their Applications to Humans. Sci Rep (2020) 10(1):9971. doi: 10.1038/s41598-020-66709-z 32561798 PMC7305326

[28] Mangoni ME Nargeot J. Genesis and Regulation of the Heart Automaticity. Physiol Rev (2008) 88(3):919–82. doi: 10.1152/physrev.00018.2007 18626064

[29] MacDonald EA Rose RA Quinn TA. Neurohumoral Control of Sinoatrial Node Activity and Heart Rate: Insight from Experimental Models and Findings from Humans. Front Physiol (2020) 11:170. doi: 10.3389/fphys.2020.00170 32194439 PMC7063087

[30] Larsson PH. How Is the Heart Rate Regulated in the Sinoatrial Node? Another Piece to the Puzzle. J Gen Physiol (2010) 136(3):237–41. 10.1085/jgp.201010506 PMC2931147 20713549

[31] Grimm M Brown JH. Beta-Adrenergic Receptor Signaling in the Heart: Role of Camkii. J Mol Cell Cardiol (2010) 48(2):322–30. doi: 10.1016/j.yjmcc.2009.10.016 PMC2896283 19883653

[32] Behar J Ganesan A Zhang J Yaniv Y. The Autonomic Nervous System Regulates the Heart Rate through Camp-Pka Dependent and Independent Coupled-Clock Pacemaker Cell Mechanisms. Front Physiol (2016) 7:419. doi: 10.3389/fphys.2016.00419 27729868 PMC5037226

[33] Vinogradova TM Zhou YY Bogdanov KY Yang D Kuschel M Cheng H. Sinoatrial Node Pacemaker Activity Requires Ca(2+)/Calmodulin-Dependent Protein Kinase Ii Activation. Circ Res (2000) 87(9):760–7. doi: 10.1161/01.res.87.9.760 11055979

[34] Wahl-Schott C Fenske S Biel M. Hcn Channels: New Roles in Sinoatrial Node Function. Curr Opin Pharmacol (2014) 15:83–90. doi: 10.1016/j.coph.2013.12.005 24441197

[35] Liao Z Lockhead D Larson ED Proenza C. Phosphorylation and Modulation of Hyperpolarization-Activated Hcn4 Channels by Protein Kinase a in the Mouse Sinoatrial Node. J Gen Physiol (2010) 136(3):247–58. doi: 10.1085/jgp.201010488 PMC2931151 20713547

[36] Hennis K Biel M Wahl-Schott C Fenske S. Beyond Pacemaking: Hcn Channels in Sinoatrial Node Function. Prog Biophys Mol Biol (2021) 166:51–60. doi: 10.1016/j.pbiomolbio.2021.03.004 33753086

[37] Yaniv Y Lakatta EG Maltsev VA. From Two Competing Oscillators to One Coupled-Clock Pacemaker Cell System. Front Physiol (2015) 6:28. doi: 10.3389/fphys.2015.00028 25741284 PMC4327306

[38] Di Francesco D. A New Interpretation of the Pace-Maker Current in Calf Purkinje Fibres. J Physiol (1981) 314:359–76. doi: 10.1113/jphysiol.1981.sp013713 PMC1249439 6273533

[39] Hagiwara N Irisawa H Kameyama M. Contribution of Two Types of Calcium Currents to the Pacemaker Potentials of Rabbit Sino-Atrial Node Cells. J Physiol (1988) 395:233–53. doi: 10.1113/jphysiol.1988.sp016916 PMC1191991 2457676

[40] Marionneau C Couette B Liu J Li H Mangoni ME Nargeot J. Specific Pattern of Ionic Channel Gene Expression Associated with Pacemaker Activity in the Mouse Heart. J Physiol (2005) 562(Pt 1):223–34. doi: 10.1113/jphysiol.2004.074047 PMC1665484 15498808

[41] Sanders L Rakovic S Lowe M Mattick PA Terrar DA. Fundamental Importance of Na+-Ca2+ Exchange for the Pacemaking Mechanism in Guinea-Pig Sino-Atrial Node. J Physiol (2006) 571(Pt 3):639–49. doi: 10.1113/jphysiol.2005.100305 PMC1805802 16423859

[42] Shibasaki T. Conductance and Kinetics of Delayed Rectifier Potassium Channels in Nodal Cells of the Rabbit Heart. J Physiol (1987) 387:227–50. doi: 10.1113/jphysiol.1987.sp016571 PMC1192502 2443680

[43] Sanguinetti MC Jurkiewicz NK. Two Components of Cardiac Delayed Rectifier K+ Current. Differential Sensitivity to Block by Class Iii Antiarrhythmic Agents. J Gen Physiol (1990) 96(1):195–215. doi: 10.1085/jgp.96.1.195 2170562 PMC2228985

[44] Rigg L Heath BM Cui Y Terrar DA. Localisation and Functional Significance of Ryanodine Receptors During Beta-Adrenoceptor Stimulation in the Guinea-Pig Sino-Atrial Node. Cardiovasc Res (2000) 48(2):254–64. doi: 10.1016/s0008-6363(00)00153-x 11054472

[45] Tsutsui K Monfredi OJ Sirenko-Tagirova SG Maltseva LA Bychkov R Kim MS. A Coupled-Clock System Drives the Automaticity of Human Sinoatrial Nodal Pacemaker Cells. Sci Signal (2018) 11(534):1–27. doi: 10.1126/scisignal.aap7608 PMC6138244 29895616

[46] Sirenko ST Zahanich I Li Y Lukyanenko YO Lyashkov AE Ziman BD. Phosphoprotein Phosphatase 1 but Not 2a Activity Modulates Coupled-Clock Mechanisms to Impact on Intrinsic Automaticity of Sinoatrial Nodal Pacemaker Cells. Cells (2021) 10(11):1–21. doi: 10.3390/cells10113106 PMC8623309 34831329

[47] Boles A Kandimalla R Reddy PH. Dynamics of Diabetes and Obesity: Epidemiological Perspective. Biochim Biophys Acta Mol Basis Dis (2017) 1863(5):1026–36. doi: 10.1016/j.bbadis.2017.01.016 PMC5429876 28130199

[48] Atlas ID. Diabetes around the World in 2021. In: International Diabetes Federation 10th Edition.

[49] Pastore I Bolla AM Montefusco L Lunati ME Rossi A Assi E. The Impact of Diabetes Mellitus on Cardiovascular Risk Onset in Children and Adolescents. Int J Mol Sci (2020) 21(14):1–17. doi: 10.3390/ijms21144928 PMC7403998 32664699

[50] Tancredi M Rosengren A Svensson AM Kosiborod M Pivodic A Gudbjornsdottir S. Excess Mortality among Persons with Type 2 Diabetes. N Engl J Med (2015) 373(18):1720–32. doi: 10.1056/NEJMoa1504347 26510021

[51] Assoumou HG Pichot V Barthelemy JC Dauphinot V Celle S Gosse P. Metabolic Syndrome and Short-Term and Long-Term Heart Rate Variability in Elderly Free of Clinical Cardiovascular Disease: The Proof Study. Rejuvenation Res (2010) 13(6):653–63. doi: 10.1089/rej.2010.1019 20818933

[52] Lin YK Chen YJ Chen SA. Potential Atrial Arrhythmogenicity of Adipocytes: Implications for the Genesis of Atrial Fibrillation. Med Hypotheses (2010) 74(6):1026–9. doi: 10.1016/j.mehy.2010.01.004 20149554

[53] Sornelli F Fiore M Chaldakov GN Aloe L. Adipose Tissue-Derived Nerve Growth Factor and Brain-Derived Neurotrophic Factor: Results from Experimental Stress and Diabetes. Gen Physiol Biophys (2009) 28:179–83. 19893098

[54] Soltysinska E Speerschneider T Winther SV Thomsen MB. Sinoatrial Node Dysfunction Induces Cardiac Arrhythmias in Diabetic Mice. Cardiovasc Diabetol (2014) 13:122. doi: 10.1186/s12933-014-0122-y 25113792 PMC4149194

[55] Vinogradova TM Bogdanov KY Lakatta EG. Beta-Adrenergic Stimulation Modulates Ryanodine Receptor Ca(2+) Release During Diastolic Depolarization to Accelerate Pacemaker Activity in Rabbit Sinoatrial Nodal Cells. Circ Res (2002) 90(1):73–9. doi: 10.1161/hh0102.102271 11786521

[56] Deng W Bukiya AN Rodriguez-Menchaca AA Zhang Z Baumgarten CM Logothetis DE. Hypercholesterolemia Induces up-Regulation of Kach Cardiac Currents *Via* a Mechanism Independent of Phosphatidylinositol 4,5-Bisphosphate and Gbetagamma. J Biol Chem (2012) 287(7):4925–35. doi: 10.1074/jbc.M111.306134 PMC3281659 22174416

[57] Singh JP Larson MG O'Donnell CJ Wilson PF Tsuji H Lloyd-Jones DM. Association of Hyperglycemia with Reduced Heart Rate Variability (the Framingham Heart Study). Am J Cardiol (2000) 86(3):309–12. doi: 10.1016/s0002-9149(00)00920-6 10922439

[58] Liu Y Jansen HJ Krishnaswamy PS Bogachev O Rose RA. Impaired Regulation of Heart Rate and Sinoatrial Node Function by the Parasympathetic Nervous System in Type 2 Diabetic Mice. Sci Rep (2021) 11(1):12465. doi: 10.1038/s41598-021-91937-2 34127743 PMC8203800

[59] Bakkar NZ Dwaib HS Fares S Eid AH Al-Dhaheri Y El-Yazbi AF. Cardiac Autonomic Neuropathy: A Progressive Consequence of Chronic Low-Grade Inflammation in Type 2 Diabetes and Related Metabolic Disorders. Int J Mol Sci (2020) 21(23):1–20. doi: 10.3390/ijms21239005 PMC7730941 33260799

[60] Vinik AI Ziegler D. Diabetic Cardiovascular Autonomic Neuropathy. Circulation (2007) 115(3):387–97. doi: 10.1161/CIRCULATIONAHA.106.634949 17242296

[61] Maser RE Mitchell BD Vinik AI Freeman R. The Association between Cardiovascular Autonomic Neuropathy and Mortality in Individuals with Diabetes: A Meta-Analysis. Diabetes Care (2003) 26(6):1895–901. doi: 10.2337/diacare.26.6.1895 12766130

[62] Kahn SE Cooper ME Del Prato S. Pathophysiology and Treatment of Type 2 Diabetes: Perspectives on the Past, Present, and Future. Lancet (2014) 383(9922):1068–83. doi: 10.1016/S0140-6736(13)62154-6 PMC4226760 24315620

[63] Krishnaswamy PS Egom EE Moghtadaei M Jansen HJ Azer J Bogachev O. Altered Parasympathetic Nervous System Regulation of the Sinoatrial Node in Akita Diabetic Mice. J Mol Cell Cardiol (2015) 82:125–35. doi: 10.1016/j.yjmcc.2015.02.024 25754673

[64] Park HJ Zhang Y Du C Welzig CM Madias C Aronovitz MJ. Role of Srebp-1 in the Development of Parasympathetic Dysfunction in the Hearts of Type 1 Diabetic Akita Mice. Circ Res (2009) 105(3):287–94. doi: 10.1161/CIRCRESAHA.109.193995 PMC2730600 19423844

[65] Cseh D Climie RE Offredo L Guibout C Thomas F Zanoli L. Type 2 Diabetes Mellitus Is Independently Associated with Decreased Neural Baroreflex Sensitivity: The Paris Prospective Study Iii. Arterioscler Thromb Vasc Biol (2020) 40(5):1420–8. doi: 10.1161/ATVBAHA.120.314102 32188272

[66] Ruiz J Monbaron D Parati G Perret S Haesler E Danzeisen C. Diabetic Neuropathy Is a More Important Determinant of Baroreflex Sensitivity Than Carotid Elasticity in Type 2 Diabetes. Hypertension (2005) 46(1):162–7. doi: 10.1161/01.HYP.0000169053.14440.7d 15928031

[67] Ziegler D Zentai CP Perz S Rathmann W Haastert B Doring A. Prediction of Mortality Using Measures of Cardiac Autonomic Dysfunction in the Diabetic and Nondiabetic Population: The Monica/Kora Augsburg Cohort Study. Diabetes Care (2008) 31(3):556–61. doi: 10.2337/dc07-1615 18086873

[68] Ewing DJ Martyn CN Young RJ Clarke BF. The Value of Cardiovascular Autonomic Function Tests: 10 Years Experience in Diabetes. Diabetes Care (1985) 8(5):491–8. doi: 10.2337/diacare.8.5.491 4053936

[69] Boudet G Walther G Courteix D Obert P Lesourd B Pereira B. Paradoxical Dissociation between Heart Rate and Heart Rate Variability Following Different Modalities of Exercise in Individuals with Metabolic Syndrome: The Resolve Study. Eur J Prev Cardiol (2017) 24(3):281–96. doi: 10.1177/2047487316679523 27856807

[70] Schroeder EB Liao D Chambless LE Prineas RJ Evans GW Heiss G. Hypertension, Blood Pressure, and Heart Rate Variability: The Atherosclerosis Risk in Communities (Aric) Study. Hypertension (2003) 42(6):1106–11. doi: 10.1161/01.HYP.0000100444.71069.73 14581296

[71] Hufnagel C Chambres P Bertrand PR Dutheil F. The Need for Objective Measures of Stress in Autism. Front Psychol (2017) 8:64. doi: 10.3389/fpsyg.2017.00064 28191002 PMC5269614

[72] Dutheil F Chambres P Hufnagel C Auxiette C Chausse P Ghozi R. 'Do Well B.': Design of Well Being Monitoring Systems. A Study Protocol for the Application in Autism. BMJ Open (2015) 5(2):e007716. doi: 10.1136/bmjopen-2015-007716 PMC4336464 25710916

[73] de Andrade PE do Amaral JAT Paiva LDS Adami F Raimudo JZ Valenti VE. Reduction of Heart Rate Variability in Hypertensive Elderly. Blood Press (2017) 26(6):350–8. doi: 10.1080/08037051.2017.1354285 28738697

[74] Yadav RL Yadav PK Yadav LK Agrawal K Sah SK Islam MN. Association between Obesity and Heart Rate Variability Indices: An Intuition toward Cardiac Autonomic Alteration - a Risk of Cvd. Diabetes Metab Syndr Obes (2017) 10:57–64. doi: 10.2147/DMSO.S123935 28255249 PMC5322847

[75] Karason K Molgaard H Wikstrand J Sjostrom L. Heart Rate Variability in Obesity and the Effect of Weight Loss. Am J Cardiol (1999) 83(8):1242–7. doi: 10.1016/s0002-9149(99)00066-1 10215292

[76] Solanki JD Basida SD Mehta HB Panjwani SJ Gadhavi BP. Comparative Study of Cardiac Autonomic Status by Heart Rate Variability between under-Treatment Normotensive and Hypertensive Known Type 2 Diabetics. Indian Heart J (2017) 69(1):52–6. doi: 10.1016/j.ihj.2016.07.013 PMC5319128 28228307

[77] Lotric MB Stefanovska A Stajer D Urbancic-Rovan V. Spectral Components of Heart Rate Variability Determined by Wavelet Analysis. Physiol Meas (2000) 21(4):441–57. doi: 10.1088/0967-3334/21/4/302 11110243

[78] Jaiswal M Urbina EM Wadwa RP Talton JW D'Agostino RB Jr. Hamman RF. Reduced Heart Rate Variability among Youth with Type 1 Diabetes: The Search Cvd Study. Diabetes Care (2013) 36(1):157–62. doi: 10.2337/dc12-0463 PMC3526238 22961570

[79] Shepard PD Canavier CC Levitan ES. Ether-a-Go-Go-Related Gene Potassium Channels: What's All the Buzz About? Schizophr Bull (2007) 33(6):1263–9. doi: 10.1093/schbul/sbm106 PMC2779881 17905786

[80] Lomax AE Rose RA Giles WR. Electrophysiological Evidence for a Gradient of G Protein-Gated K+ Current in Adult Mouse Atria. Br J Pharmacol (2003) 140(3):576–84. doi: 10.1038/sj.bjp.0705474 PMC1574060 14522844

[81] Shui Z Boyett MR Zang WJ Haga T Kameyama K. Receptor Kinase-Dependent Desensitization of the Muscarinic K+ Current in Rat Atrial Cells. J Physiol (1995) 487(Pt 2):359–66. doi: 10.1113/jphysiol.1995.sp020885 PMC1156578 8558469

[82] Bender K Wellner-Kienitz MC Bosche LI Rinne A Beckmann C Pott L. Acute Desensitization of Girk Current in Rat Atrial Myocytes Is Related to K+ Current Flow. J Physiol (2004) 561(Pt 2):471–83. doi: 10.1113/jphysiol.2004.072462 PMC1665358 15459243

[83] Cifelli C Rose RA Zhang H Voigtlaender-Bolz J Bolz SS Backx PH. Rgs4 Regulates Parasympathetic Signaling and Heart Rate Control in the Sinoatrial Node. Circ Res (2008) 103(5):527–35. doi: 10.1161/CIRCRESAHA.108.180984 18658048

[84] Ishii M Inanobe A Kurachi Y. Pip3 Inhibition of Rgs Protein and Its Reversal by Ca2+/Calmodulin Mediate Voltage-Dependent Control of the G Protein Cycle in a Cardiac K+ Channel. Proc Natl Acad Sci USA (2002) 99(7):4325–30. doi: 10.1073/pnas.072073399 PMC123647 11904384

[85] Oudit GY Sun H Kerfant BG Crackower MA Penninger JM Backx PH. The Role of Phosphoinositide-3 Kinase and Pten in Cardiovascular Physiology and Disease. J Mol Cell Cardiol (2004) 37(2):449–71. doi: 10.1016/j.yjmcc.2004.05.015 15276015

[86] Bertrand L Horman S Beauloye C Vanoverschelde JL. Insulin Signalling in the Heart. Cardiovasc Res (2008) 79(2):238–48. doi: 10.1093/cvr/cvn093 18390897

[87] Swaminathan PD Purohit A Soni S Voigt N Singh MV Glukhov AV. Oxidized Camkii Causes Cardiac Sinus Node Dysfunction in Mice. J Clin Invest (2011) 121(8):3277–88. doi: 10.1172/JCI57833 PMC3223923 21785215

[88] Luo M Guan X Luczak ED Lang D Kutschke W Gao Z. Diabetes Increases Mortality after Myocardial Infarction by Oxidizing Camkii. J Clin Invest (2013) 123(3):1262–74. doi: 10.1172/JCI65268 PMC3673230 23426181

[89] Wu Y Anderson ME. Camkii in Sinoatrial Node Physiology and Dysfunction. Front Pharmacol (2014) 5:48. doi: 10.3389/fphar.2014.00048 24672485 PMC3957193

[90] Volpe CMO Villar-Delfino PH Dos Anjos PMF Nogueira-Machado JA. Cellular Death, Reactive Oxygen Species (Ros) and Diabetic Complications. Cell Death Dis (2018) 9(2):119. doi: 10.1038/s41419-017-0135-z 29371661 PMC5833737

[91] Vinogradova T Tarasov K Riordon D Tarasova Y Lakatta E. Normal Spontaneous Firing of Cardiac Pacemaker Cells Is Regulated by Basal Pkc Delta Activation. Eur Heart J (2020) 41.

[92] Kondo H Kira S Oniki T Gotoh K Fukui A Abe I. Interleukin-10 Treatment Attenuates Sinus Node Dysfunction Caused by Streptozotocin-Induced Hyperglycaemia in Mice. Cardiovasc Res (2019) 115(1):57–70. doi: 10.1093/cvr/cvy162 29982291

[93] Levelt E Pavlides M Banerjee R Mahmod M Kelly C Sellwood J. Ectopic and Visceral Fat Deposition in Lean and Obese Patients with Type 2 Diabetes. J Am Coll Cardiol (2016) 68(1):53–63. doi: 10.1016/j.jacc.2016.03.597 27364051 PMC4925621

[94] Yanni J Tellez JO Sutyagin PV Boyett MR Dobrzynski H. Structural Remodelling of the Sinoatrial Node in Obese Old Rats. J Mol Cell Cardiol (2010) 48(4):653–62. doi: 10.1016/j.yjmcc.2009.08.023 PMC2845824 19729016

[95] Grden M Podgorska M Szutowicz A Pawelczyk T. Altered Expression of Adenosine Receptors in Heart of Diabetic Rat. J Physiol Pharmacol (2005) 56(4):587–97. 16391416

[96] Lou Q Hansen BJ Fedorenko O Csepe TA Kalyanasundaram A Li N. Upregulation of Adenosine A1 Receptors Facilitates Sinoatrial Node Dysfunction in Chronic Canine Heart Failure by Exacerbating Nodal Conduction Abnormalities Revealed by Novel Dual-Sided Intramural Optical Mapping. Circulation (2014) 130(4):315–24. doi: 10.1161/CIRCULATIONAHA.113.007086 PMC4323163 24838362

[97] Gallego M Zayas-Arrabal J Alquiza A Apellaniz B Casis O. Electrical Features of the Diabetic Myocardium. Arrhythmic and Cardiovascular Safety Considerations in Diabetes. Front Pharmacol (2021) 12:687256. doi: 10.3389/fphar.2021.687256 34305599 PMC8295895

[98] Siscovick DS Sotoodehnia N Rea TD Raghunathan TE Jouven X Lemaitre RN. Type 2 Diabetes Mellitus and the Risk of Sudden Cardiac Arrest in the Community. Rev Endocr Metab Disord (2010) 11(1):53–9. doi: 10.1007/s11154-010-9133-5 PMC3413310 20195771

[99] Wasada T Katsumori K Hasumi S Kasanuki H Arii H Saeki A. Association of Sick Sinus Syndrome with Hyperinsulinemia and Insulin Resistance in Patients With Non-Insulin-Dependent Diabetes Mellitus: Report of Four Cases. Intern Med (1995) 34(12):1174–7. doi: 10.2169/internalmedicine.34.1174 8929644

[100] Guzman E Singh N Khan IA Niarchos AP Verghese C Saponieri C. Left Bundle Branch Block in Type 2 Diabetes Mellitus: A Sign of Advanced Cardiovascular Involvement. Ann Noninvasive Electrocardiol (2004) 9(4):362–5. doi: 10.1111/j.1542-474X.2004.94577.x PMC6932130 15485515

[101] Movahed MR Hashemzadeh M Jamal MM. Increased Prevalence of Third-Degree Atrioventricular Block in Patients with Type II Diabetes Mellitus. Chest (2005) 128(4):2611–4. doi: 10.1378/chest.128.4.2611 16236932

[102] Lychev VG Klester EB Plinokosova LA. Arrhythmia in Patients with Chronic Heart Insufficiency and Type 2 Diabetes Mellitus. Klin Med (Mosk) (2014) 92(3):38–42. 25269194

[103] Huxley RR Filion KB Konety S Alonso A. Meta-Analysis of Cohort and Case-Control Studies of Type 2 Diabetes Mellitus and Risk of Atrial Fibrillation. Am J Cardiol (2011) 108(1):56–62. doi: 10.1016/j.amjcard.2011.03.004 21529739 PMC3181495

[104] Hillis GS Woodward M Rodgers A Chow CK Li Q Zoungas S. Resting Heart Rate and the Risk of Death and Cardiovascular Complications in Patients with Type 2 Diabetes Mellitus. Diabetologia (2012) 55(5):1283–90. doi: 10.1007/s00125-012-2471-y PMC4170780 22286552

[105] Movahed MR Hashemzadeh M Jamal MM. Increased Prevalence of Infectious Endocarditis in Patients With Type II Diabetes Mellitus. J Diabetes Complications (2007) 21(6):403–6. doi: 10.1016/j.jdiacomp.2007.07.003 17967715

[106] Howarth FC Jacobson M Shafiullah M Adeghate E. Long-Term Effects of Type 2 Diabetes Mellitus on Heart Rhythm in the Goto-Kakizaki Rat. Exp Physiol (2008) 93(3):362–9. doi: 10.1113/expphysiol.2007.040055 18156165

[107] Ferdous Z Qureshi MA Jayaprakash P Parekh K John A Oz M. Different Profile of Mrna Expression in Sinoatrial Node from Streptozotocin-Induced Diabetic Rat. PloS One (2016) 11(4):e0153934. doi: 10.1371/journal.pone.0153934 27096430 PMC4838258

[108] Lengyel C Virag L Biro T Jost N Magyar J Biliczki P. Diabetes Mellitus Attenuates the Repolarization Reserve in Mammalian Heart. Cardiovasc Res (2007) 73(3):512–20. doi: 10.1016/j.cardiores.2006.11.010 17182020

[109] Torres-Jacome J Gallego M Rodriguez-Robledo JM Sanchez-Chapula JA Casis O. Improvement of the Metabolic Status Recovers Cardiac Potassium Channel Synthesis in Experimental Diabetes. Acta Physiol (Oxf) (2013) 207(3):447–59. doi: 10.1111/apha.12043 23181465

[110] Huang X Zhong N Zhang H Ma A Yuan Z Guo N. Reduced Expression of Hcn Channels in the Sinoatrial Node of Streptozotocin-Induced Diabetic Rats. Can J Physiol Pharmacol (2017) 95(5):586–94. doi: 10.1139/cjpp-2016-0418 28177679

[111] Shinagawa Y Satoh H Noma A. The Sustained Inward Current and Inward Rectifier K+ Current in Pacemaker Cells Dissociated from Rat Sinoatrial Node. J Physiol (2000) 523 Pt 3:593–605. doi: 10.1111/j.1469-7793.2000.t01-2-00593.x 10718740 PMC2269831

[112] Nof E Vysochek L Meisel E Burashnikov E Antzelevitch C Clatot J. Mutations in Nav1.5 Reveal Calcium-Calmodulin Regulation of Sodium Channel. Front Physiol (2019) 10:700. doi: 10.3389/fphys.2019.00700 31231243 PMC6560087

[113] Lei M Jones SA Liu J Lancaster MK Fung SS Dobrzynski H. Requirement of Neuronal- and Cardiac-Type Sodium Channels for Murine Sinoatrial Node Pacemaking. J Physiol (2004) 559(Pt 3):835–48. doi: 10.1113/jphysiol.2004.068643 PMC1665172 15254155

[114] Protas L Oren RV Clancy CE Robinson RB. Age-Dependent Changes in Na Current Magnitude and Ttx-Sensitivity in the Canine Sinoatrial Node. J Mol Cell Cardiol (2010) 48(1):172–80. doi: 10.1016/j.yjmcc.2009.07.028 PMC2813407 19665465

[115] Li N Kalyanasundaram A Hansen BJ Artiga EJ Sharma R Abudulwahed SH. Impaired Neuronal Sodium Channels Cause Intranodal Conduction Failure and Reentrant Arrhythmias in Human Sinoatrial Node. Nat Commun (2020) 11(1):512. doi: 10.1038/s41467-019-14039-8 31980605 PMC6981137

[116] Papadatos GA Wallerstein PM Head CE Ratcliff R Brady PA Benndorf K. Slowed Conduction and Ventricular Tachycardia after Targeted Disruption of the Cardiac Sodium Channel Gene Scn5a. Proc Natl Acad Sci USA (2002) 99(9):6210–5. doi: 10.1073/pnas.082121299 PMC122928 11972032

[117] Stables CL Musa H Mitra A Bhushal S Deo M Guerrero-Serna G. Reduced Na(+) Current Density Underlies Impaired Propagation in the Diabetic Rabbit Ventricle. J Mol Cell Cardiol (2014) 69:24–31. doi: 10.1016/j.yjmcc.2013.12.031 24412579 PMC4066653

[118] Zhang Y Wang Y Yanni J Qureshi MA Logantha S Kassab S. Electrical Conduction System Remodeling in Streptozotocin-Induced Diabetes Mellitus Rat Heart. Front Physiol (2019) 10:826. doi: 10.3389/fphys.2019.00826 31338036 PMC6628866

[119] Giacco F Brownlee M. Oxidative Stress and Diabetic Complications. Circ Res (2010) 107(9):1058–70. doi: 10.1161/CIRCRESAHA.110.223545 PMC2996922 21030723

[120] Liu M Liu H Dudley SC Jr.. Reactive Oxygen Species Originating from Mitochondria Regulate the Cardiac Sodium Channel. Circ Res (2010) 107(8):967–74. doi: 10.1161/CIRCRESAHA.110.220673 PMC2955818 20724705

[121] Chandler NJ Greener ID Tellez JO Inada S Musa H Molenaar P. Molecular Architecture of the Human Sinus Node: Insights into the Function of the Cardiac Pacemaker. Circulation (2009) 119(12):1562–75. doi: 10.1161/CIRCULATIONAHA.108.804369 19289639

[122] Fenske S Hennis K Rotzer RD Brox VF Becirovic E Scharr A. Camp-Dependent Regulation of Hcn4 Controls the Tonic Entrainment Process in Sinoatrial Node Pacemaker Cells. Nat Commun (2020) 11(1):5555. doi: 10.1038/s41467-020-19304-9 33144559 PMC7641277

[123] Zagotta WN Olivier NB Black KD Young EC Olson R Gouaux E. Structural Basis for Modulation and Agonist Specificity of Hcn Pacemaker Channels. Nature (2003) 425(6954):200–5. doi: 10.1038/nature01922 12968185

[124] DiFrancesco D Tortora P. Direct Activation of Cardiac Pacemaker Channels by Intracellular Cyclic Amp. Nature (1991) 351(6322):145–7. doi: 10.1038/351145a0 1709448

[125] Herrmann S Stieber J Ludwig A. Pathophysiology of Hcn Channels. Pflugers Arch (2007) 454(4):517–22. doi: 10.1007/s00424-007-0224-4 17549513

[126] Howarth FC Qureshi MA Jayaprakash P Parekh K Oz M Dobrzynski H. The Pattern of Mrna Expression Is Changed in Sinoatrial Node from Goto-Kakizaki Type 2 Diabetic Rat Heart. J Diabetes Res (2018) 2018:8454078. doi: 10.1155/2018/8454078 30246030 PMC6139199

[127] Grisanti LA. Diabetes and Arrhythmias: Pathophysiology, Mechanisms and Therapeutic Outcomes. Front Physiol (2018) 9:1669. doi: 10.3389/fphys.2018.01669 30534081 PMC6275303

[128] Iop L Iliceto S Civieri G Tona F. Inherited and Acquired Rhythm Disturbances in Sick Sinus Syndrome, Brugada Syndrome, and Atrial Fibrillation: Lessons from Preclinical Modeling. Cells (2021) 10(11):1–52. doi: 10.3390/cells10113175 PMC8623957 34831398

[129] Yang B Huang Y Zhang H Huang Y Zhou HJ Young L. Mitochondrial Thioredoxin-2 Maintains Hcn4 Expression and Prevents Oxidative Stress-Mediated Sick Sinus Syndrome. J Mol Cell Cardiol (2020) 138:291–303. doi: 10.1016/j.yjmcc.2019.10.009 31751569

[130] Li Y Wang F Zhang X Qi Z Tang M Szeto C. Beta-Adrenergic Stimulation Increases Cav3.1 Activity in Cardiac Myocytes through Protein Kinase A. PloS One (2012) 7(7):e39965. doi: 10.1371/journal.pone.0039965 22808078 PMC3396630

[131] Mesirca P Torrente AG. Mangoni ME. T-Type Channels in the Sino-Atrial and Atrioventricular Pacemaker Mechanism. Pflugers Arch (2014) 466(4):791–9. doi: 10.1007/s00424-014-1482-6 24573175

[132] Mangoni ME Traboulsie A Leoni AL Couette B Marger L Le Quang K. Bradycardia and Slowing of the Atrioventricular Conduction in Mice Lacking Cav3.1/Alpha1g T-Type Calcium Channels. Circ Res (2006) 98(11):1422–30. doi: 10.1161/01.RES.0000225862.14314.49 16690884

[133] Salem KA Qureshi MA Sydorenko V Parekh K Jayaprakash P Iqbal T. Effects of Exercise Training on Excitation-Contraction Coupling and Related Mrna Expression in Hearts of Goto-Kakizaki Type 2 Diabetic Rats. Mol Cell Biochem (2013) 380(1-2):83–96. doi: 10.1007/s11010-013-1662-2 23620341

[134] Howarth FC Qureshi MA Hassan Z Isaev D Parekh K John A. Contractility of Ventricular Myocytes Is Well Preserved Despite Altered Mechanisms of Ca2+ Transport and a Changing Pattern of Mrna in Aged Type 2 Zucker Diabetic Fatty Rat Heart. Mol Cell Biochem (2012) 361(1-2):267–80. doi: 10.1007/s11010-011-1112-y 22009485

[135] Mangoni ME Couette B Bourinet E Platzer J Reimer D Striessnig J. Functional Role of L-Type Cav1.3 Ca2^+^ Channels in Cardiac Pacemaker Activity. Proc Natl Acad Sci USA (2003) 100(9):5543–8. doi: 10.1073/pnas.0935295100 PMC154381 12700358

[136] Torrente AG Mesirca P Neco P Rizzetto R Dubel S Barrere C. L-Type Cav1.3 Channels Regulate Ryanodine Receptor-Dependent Ca2^+^ Release During Sino-Atrial Node Pacemaker Activity. Cardiovasc Res (2016) 109(3):451–61. doi: 10.1093/cvr/cvw006 26786159

[137] Striessnig J Grabner M Mitterdorfer J Hering S Sinnegger MJ Glossmann H. Structural Basis of Drug Binding to L Ca2+ Channels. Trends Pharmacol Sci (1998) 19(3):108–15. doi: 10.1016/s0165-6147(98)01171-7 9584627

[138] Ozturk N Uslu S Ozdemir S. Diabetes-Induced Changes in Cardiac Voltage-Gated Ion Channels. World J Diabetes (2021) 12(1):1–18. doi: 10.4239/wjd.v12.i1.1 33520105 PMC7807254

[139] Factor SM Robinson TF Dominitz R Cho SH. Alterations of the Myocardial Skeletal Framework in Acute Myocardial Infarction with and without Ventricular Rupture. A Preliminary Report. Am J Cardiovasc Pathol (1987) 1(1):91–7. 2458117

[140] Ward ML Crossman DJ. Mechanisms Underlying the Impaired Contractility of Diabetic Cardiomyopathy. World J Cardiol (2014) 6(7):577–84. doi: 10.4330/wjc.v6.i7.577 PMC4110606 25068018

[141] He M Qiu J Wang Y Bai Y Chen G. Caveolin-3 and Arrhythmias: Insights into the Molecular Mechanisms. J Clin Med (2022) 11(6):1–13. doi: 10.3390/jcm11061595 PMC8952412 35329921

[142] Parton RG. Caveolae: Structure, Function, and Relationship to Disease. Annu Rev Cell Dev Biol (2018) 34:111–36. doi: 10.1146/annurev-cellbio-100617-062737 30296391

[143] Koga A Oka N Kikuchi T Miyazaki H Kato S Imaizumi T. Adenovirus-Mediated Overexpression of Caveolin-3 Inhibits Rat Cardiomyocyte Hypertrophy. Hypertension (2003) 42(2):213–9. doi: 10.1161/01.HYP.0000082926.08268.5D 12847114

[144] Lang D Warden A Balijepalli R Kamp TJ Glukhov AV. Loss of Caveolin-3 Disrupts Mouse Sinoatrial Node Pacemaking and Stimulates Atrial Arrhythmogenesis. Circulation (2016) 134:A15361. doi: 10.1161/circ.134.suppl_1.15361

[145] Barbuti A Terragni B Brioschi C DiFrancesco D. Localization of F-Channels to Caveolae Mediates Specific Beta2-Adrenergic Receptor Modulation of Rate in Sinoatrial Myocytes. J Mol Cell Cardiol (2007) 42(1):71–8. doi: 10.1016/j.yjmcc.2006.09.018 17070839

[146] Ye B Balijepalli RC Foell JD Kroboth S Ye Q Luo YH. Caveolin-3 Associates with and Affects the Function of Hyperpolarization-Activated Cyclic Nucleotide-Gated Channel 4. Biochemistry (2008) 47(47):12312–8. 10.1021/bi8009295 PMC2803323 19238754

[147] Campostrini G Bonzanni M Lissoni A Bazzini C Milanesi R Vezzoli E. The Expression of the Rare Caveolin-3 Variant T78m Alters Cardiac Ion Channels Function and Membrane Excitability. Cardiovasc Res (2017) 113(10):1256–65. doi: 10.1093/cvr/cvx122 PMC5852518 28898996

[148] Penumathsa SV Thirunavukkarasu M Zhan L Maulik G Menon VP Bagchi D. Resveratrol Enhances Glut-4 Translocation to the Caveolar Lipid Raft Fractions through Ampk/Akt/Enos Signalling Pathway in Diabetic Myocardium. J Cell Mol Med (2008) 12(6A):2350–61. doi: 10.1111/j.1582-4934.2008.00251.x PMC4514113 18266981

[149] Penumathsa SV Thirunavukkarasu M Samuel SM Zhan L Maulik G Bagchi M. Niacin Bound Chromium Treatment Induces Myocardial Glut-4 Translocation and Caveolar Interaction *Via* Akt, Ampk and Enos Phosphorylation in Streptozotocin Induced Diabetic Rats after Ischemia-Reperfusion Injury. Biochim Biophys Acta (2009) 1792(1):39–48. doi: 10.1016/j.bbadis.2008.10.018 19027847

[150] Lei S Li H Xu J Liu Y Gao X Wang J. Hyperglycemia-Induced Protein Kinase C Beta2 Activation Induces Diastolic Cardiac Dysfunction in Diabetic Rats by Impairing Caveolin-3 Expression and Akt/Enos Signaling. Diabetes (2013) 62(7):2318–28. doi: 10.2337/db12-1391 PMC3712061 23474486

[151] Verheijck EE van Kempen MJ Veereschild M Lurvink J Jongsma HJ Bouman LN. Electrophysiological Features of the Mouse Sinoatrial Node in Relation to Connexin Distribution. Cardiovasc Res (2001) 52(1):40–50. doi: 10.1016/s0008-6363(01)00364-9 11557232

[152] Joshi MS Mihm MJ Cook AC Schanbacher BL Bauer JA. Alterations in Connexin 43 During Diabetic Cardiomyopathy: Competition of Tyrosine Nitration Versus Phosphorylation. J Diabetes (2015) 7(2):250–9. doi: 10.1111/1753-0407.12164 PMC4221578 24796789

[153] Mayama T Matsumura K Lin H Ogawa K Imanaga I. Remodelling of Cardiac Gap Junction Connexin 43 and Arrhythmogenesis. Exp Clin Cardiol (2007) 12(2):67–76. 18650985 PMC2359605

[154] Howarth FC Nowotny N Zilahi E El Haj MA Lei M. Altered Expression of Gap Junction Connexin Proteins May Partly Underlie Heart Rhythm Disturbances in the Streptozotocin-Induced Diabetic Rat Heart. Mol Cell Biochem (2007) 305(1-2):145–51. doi: 10.1007/s11010-007-9537-z 17632690

[155] Brown NJ. Cardiovascular Effects of Antidiabetic Agents: Focus on Blood Pressure Effects of Incretin-Based Therapies. J Am Soc Hypertens (2012) 6(3):163–8. doi: 10.1016/j.jash.2012.02.003 PMC3422131 22433315

[156] Nantsupawat T Wongcharoen W Chattipakorn SC Chattipakorn N. Effects of Metformin on Atrial and Ventricular Arrhythmias: Evidence from Cell to Patient. Cardiovasc Diabetol (2020) 19(1):198. doi: 10.1186/s12933-020-01176-4 33234131 PMC7687769

[157] Harada M Tadevosyan A Qi X Xiao J Liu T Voigt N. Atrial Fibrillation Activates Amp-Dependent Protein Kinase and Its Regulation of Cellular Calcium Handling: Potential Role in Metabolic Adaptation and Prevention of Progression. J Am Coll Cardiol (2015) 66(1):47–58. doi: 10.1016/j.jacc.2015.04.056 26139058

[158] Dart C. Selective Block of K(Atp) Channels: Why the Anti-Diabetic Sulphonylureas and Rosiglitazone Have More in Common Than We Thought. Br J Pharmacol (2012) 167(1):23–5. doi: 10.1111/j.1476-5381.2012.01990.x PMC3448910 22506686

[159] Ashcroft FM Gribble FM. Atp-Sensitive K+ Channels and Insulin Secretion: Their Role in Health and Disease. Diabetologia (1999) 42(8):903–19. doi: 10.1007/s001250051247 10491749

[160] Lu L Reiter MJ Xu Y Chicco A Greyson CR Schwartz GG. Thiazolidinedione Drugs Block Cardiac Katp Channels and May Increase Propensity for Ischaemic Ventricular Fibrillation in Pigs. Diabetologia (2008) 51(4):675–85. doi: 10.1007/s00125-008-0924-0 PMC3633423 18251006

[161] Yu L Jin X Yang Y Cui N Jiang C. Rosiglitazone Inhibits Vascular Katp Channels and Coronary Vasodilation Produced by Isoprenaline. Br J Pharmacol (2011) 164(8):2064–72. doi: 10.1111/j.1476-5381.2011.01539.x PMC3246668 21671900

[162] Bonnet F Scheen AJ. Impact of Glucose-Lowering Therapies on Risk of Stroke in Type 2 Diabetes. Diabetes Metab (2017) 43(4):299–313. doi: 10.1016/j.diabet.2017.04.004 28522196

[163] Shimoni Y Ewart HS Severson D. Insulin Stimulation of Rat Ventricular K+ Currents Depends on the Integrity of the Cytoskeleton. J Physiol (1999) 514(Pt 3):735–45. doi: 10.1111/j.1469-7793.1999.735ad.x PMC2269091 9882746

[164] Aulbach F Simm A Maier S Langenfeld H Walter U Kersting U. Insulin Stimulates the L-Type Ca2+ Current in Rat Cardiac Myocytes. Cardiovasc Res (1999) 42(1):113–20. doi: 10.1016/s0008-6363(98)00307-1 10435002

[165] Shimoni Y Severson D Ewart HS. Insulin Resistance and the Modulation of Rat Cardiac K(+) Currents. Am J Physiol Heart Circ Physiol (2000) 279(2):H639–49. doi: 10.1152/ajpheart.2000.279.2.H639 10924063

[166] Polina I Jansen HJ Li T Moghtadaei M Bohne LJ Liu Y. Loss of Insulin Signaling May Contribute to Atrial Fibrillation and Atrial Electrical Remodeling in Type 1 Diabetes. Proc Natl Acad Sci USA (2020) 117(14):7990–8000. doi: 10.1073/pnas.1914853117 32198206 PMC7148583

